# Pregnancy and Birth Outcomes in Patients With Multidrug-Resistant Tuberculosis Treated With Regimens That Include New and Repurposed Drugs

**DOI:** 10.1093/cid/ciad445

**Published:** 2023-08-22

**Authors:** Ismat Lotia Farrukh, Nathalie Lachenal, Malik M Adenov, Saman Ahmed, Yerkebulan Algozhin, Sylvine Coutisson, Epifanio Sánchez Garavito, Catherine Hewison, David Holtzman, Helena Huerga, Aleeza Janmohamed, Palwasha Y Khan, Gamarly Leblanc Jacques, Nino Lomtadze, Nara Melikyan, Carole D Mitnick, Gulnaz Mussabekova, Elna Osso, Sara Perea, Fauziah Asnely Putri, Mahmud Rashidov, Michael L Rich, Yekaterina Sakhabutdinova, Kwonjune J Seung, Assel Stambekova, Dante Vargas Vásquez, Molly F Franke, Uzma Khan

**Affiliations:** Interactive Research and Development Pakistan, Karachi, Pakistan; Pharmacovigilance Unit, Médecins Sans Frontières, Geneva, Switzerland; National Scientific Center of Phthisiopulmonology, Ministry of Health, Republic of Kazakhstan, Almaty, Kazakhstan; Interactive Research and Development Pakistan, Karachi, Pakistan; Partners In Health, Almaty, Kazakhstan; Pharmacovigilance Unit, Médecins Sans Frontières, Geneva, Switzerland; Centro de Investigación de Enfermedades Neumológicas, Hospital Nacional Sergio Bernale, Lima, Peru; Medical Department, Médecins Sans Frontières, Paris, France; Partners In Health, Maseru, Lesotho; Field Epidemiology Department, Epicentre, Paris, France; Interactive Research and Development Pakistan, Karachi, Pakistan; Interactive Research and Development Pakistan, Karachi, Pakistan; London School of Hygiene & Tropical Medicine, London, United Kingdom; Zanmi Lasante, Cange, Haiti; Surveillance and Strategic Planning, Ministry of Health, Tbilisi, Georgia; Field Epidemiology Department, Epicentre, Yerevan, Armenia; Department of Global Health and Social Medicine, Harvard Medical School, Boston, Massachusetts, USA; Partners In Health, Boston, Massachusetts, USA; National Scientific Center of Phthisiopulmonology, Ministry of Health, Republic of Kazakhstan, Almaty, Kazakhstan; Department of Global Health and Social Medicine, Harvard Medical School, Boston, Massachusetts, USA; Socios En Salud Sucursal Peru, Lima, Peru; Interactive Research and Development Global, Jakarta, Indonesia; Partners In Health, Almaty, Kazakhstan; Partners In Health, Boston, Massachusetts, USA; Division of Global Health Equity, Brigham and Women's Hospital, Boston, Massachusetts, USA; Partners In Health, Almaty, Kazakhstan; Partners In Health, Boston, Massachusetts, USA; Division of Global Health Equity, Brigham and Women's Hospital, Boston, Massachusetts, USA; Partners In Health, Almaty, Kazakhstan; Centro de Investigación, Hospital Nacional Hipólito Unanue, Lima, Peru; Department of Global Health and Social Medicine, Harvard Medical School, Boston, Massachusetts, USA; Interactive Research and Development Pakistan, Karachi, Pakistan

**Keywords:** tuberculosis, drug resistance, pregnancy, outcome, treatment

## Abstract

Among 43 pregnant women receiving multidrug-resistant/rifampicin-resistant tuberculosis (MDR/RR-TB) treatment with bedaquiline and/or delamanid, 98% had favorable treatment outcomes. Of 31 continued pregnancies, 81% had live births with no reported malformations, and 68% of neonates had normal birth weights. Effective MDR/RR-TB treatment during pregnancy can improve maternal outcomes without harming neonates.

Although the prevalence of multidrug-resistant or rifampicin-resistant tuberculosis (MDR/RR-TB) among pregnant women is not well known, the global burden of TB among pregnant and post-partum women is estimated at 216 500 cases annually [1]. In resource-limited countries, the greatest burden of TB in women occurs during the reproductive years [1]. Management of MDR/RR-TB among pregnant women is complex because the potential teratogenic effects of some second-line TB medications are largely unknown due to the exclusion of pregnant and breastfeeding women from clinical trials [2, 3]. Prior to December 2022, the World Health Organization (WHO) recommended individualized treatment regimens composed of drugs with a preferred safety profile for pregnant women with MDR/RR-TB [4, 5]. The 2022 revision recommends a 9-month, all-oral regimen with linezolid instead of ethionamide for pregnant women in whom resistance to fluoroquinolones (FQs) has been excluded [5]. These recommendations are based on animal studies and limited human data from small observational studies, and the lack of data is more pronounced for drugs with recent marketing authorization such as bedaquiline and delamanid and the 9-month regimen. There is extremely limited evidence to help women, their families, and clinicians make informed decisions about MDR/RR-TB treatment during pregnancy. We describe maternal and neonatal outcomes among pregnant women who initiated routine MDR/RR-TB treatment containing new (bedaquiline and delamanid) and repurposed drugs (eg, linezolid, clofazimine).

## METHODS

### Study Design

Data on pregnancy, birth, and treatment outcomes were systematically collected by the Médecins Sans Frontières (MSF) pharmacovigilance unit (PVU) supporting 2 observational studies. The endTB observational study was a multicenter prospective cohort study enrolling patients with MDR/RR-TB who initiated treatment with new and repurposed drugs under routine care from April 2015 through September 2018 in 17 countries [6, 7]. The ongoing Strengthening Evidence on Optimal Treatment of Multidrug-Resistant Tuberculosis (STEM-TB) cohort extended the endTB observational study in 3 countries by enrolling patients receiving 9-month all-oral bedaquiline-containing regimens under operational research conditions beginning in September 2020. STEM-TB data through March 2023 were used for this analysis. All individualized treatment regimens were based on National TB Program and WHO guidelines and informed by the study-specific clinical guides to support physicians and programs in regimen design, monitoring, and management [8, 9]. For both cohorts, any patient with MDR/RR-TB who reported a pregnancy at any time during treatment or follow-up was included in this study. Pregnancy notification lasted for a maximum of 6 months from the last dose of bedaquiline. The PVU collected reports of serious adverse events, pregnancies, and medication errors. Patients were followed minimally until the end of MDR/RR-TB treatment, and pregnancies were followed until their outcome was known.

### Data Collection

Clinical, sociodemographic, and microbiological data; laboratory results; comorbidities; safety data; and TB treatment records were entered into an electronic medical record and PVU database (Basecon, Hedehusene, Denmark) [7]. Follow-up was performed according to monitoring schedules recommended by national and WHO guidelines. Clinicians assessed, recorded, and reported pregnancies to the PVU using a standardized pregnancy report form, recording estimated date of conception, drug exposure, pregnancy outcome, birth, birthweight, and neonatal malformations (minimally up to neonatal period). Treatment outcomes were defined based on WHO guidelines, with success defined as cure or treatment completion [10]. Poor treatment outcomes were defined as the sum of death, loss to follow-up, and treatment failure. Pregnancy outcomes were classified as live birth, elective termination of pregnancy, spontaneous abortion, or outcome unknown [11, 12].

### Statistical Analysis

Descriptive statistics were calculated using Stata/SE 14.2 (StataCorp, College Station, TX).

### Ethics Approval

The endTB study (NCT03259269) was approved by the Partners Healthcare Human Research Committee (Boston, Massachusetts), the MSF Ethics Review Board (Geneva, Switzerland), and the Interactive Research and Development (IRD) Institutional Review Board (Karachi, Pakistan). STEM-TB (NCT05871489) was approved by the Harvard Medical School Institutional Review Board (IRB20-1316). Both studies were approved in enrolling countries by local ethics committees. Participants provided written informed consent.

## RESULTS

Between 1 April 2015 and 1 March 2023, 1057 women of childbearing age (15–49 years) received treatment with bedaquiline and/or delamanid, and 48 pregnancies in 43 women were notified to the study (Table 1). These women came from 8 countries; their median age was 28 years (range, 18–39), and half (52%) had a normal body mass index at the time of treatment initiation. One (2%) was human immunodeficiency virus (HIV)–positive at the time of enrollment. Most women (74%) had been previously treated for TB; more than half had resistance to an FQ, a second-line injectable, or both; and 3 (7%) had extensive disease with high bacterial load and cavitation on chest X ray. Actual treatment regimens received by pregnant patients from the endTB cohort are listed in Supplementary Table 1. The majority of patients received linezolid (95%), bedaquiline (93%), and/or clofazimine (67%) during their treatment (Table 1). Some also received delamanid (33%) and second-line injectable drugs including amikacin (21%), capreomycin (12%), and kanamycin (7%). The median duration of MDR/RR-TB treatment was 609 days (range, 251–950). Nearly all women (98%) had successful treatment outcomes, with 1 death recorded.

**Table 1.** Baseline Characteristics and Pregnancy and Treatment Outcomes of Pregnant Patients Receiving Multidrug-Resistant/Rifampicin-Resistant Tuberculosis Treatment Containing Bedaquiline and/or Delamanid

Patient Characteristic	N	%
Number of pregnant patients^a^	43	100
Year of treatment initiation		
2015	3	7
2016	10	23
2017	10	23
2018	14	33
2020	2	5
2021	4	9
Country code^b^		
A	18	42
B	9	21
C	8	19
D	4	9
E	1	2
F	1	2
G	1	2
H	1	2
Age^c^		
Median age (range)	28	(18–39)
Body mass index (n = 42)^d^		
Underweight (<18.5 kg/m^2^)	13	31
Normal (18.5–24.9 kg/m^2^)	22	52
Overweight (25–29.9 kg/m^2^)	7	17
Human immunodeficiency virus status^e^		
Positive	1	2
Negative	42	98
Treatment history		
No prior tuberculosis treatment	11	26
Previously treated	32	74
Resistance profile^f^		
MDR/RR	13	30
MDR/RR + SLI	5	12
MDR/RR + FQ	10	23
XDR	15	35
Extensive disease^g^		
Yes	3	7
No	28	65
Unknown	12	28
Treatment received		
Linezolid	41	95
Bedaquiline	40	93
Clofazimine	29	67
Delamanid	14	33
Amikacin	9	21
Capreomycin	5	12
Kanamycin	3	7
Treatment duration		
Median number of days (range)	609	251–950
Treatment outcomes^h^		
Successful	42	98
Poor	1	2

Pregnancy characteristics	N	%
Number of pregnancies^a^	48	100
Pregnancy timeline		
Before treatment initiation	8	17
During treatment	35	73
After end of treatment	5	10
Duration of treatment during pregnancy (n = 40)^i^		
Median number of days (range)	82	(9–304)
Pregnancy outcomes		
Pregnancy terminated electively	17	35
Pregnancy continued	31	65
Birth outcomes (n = 32)^j^		
Live birth	26	81
Spontaneous abortion	2	6
Unknown	4	13
Birthweight (n = 22)^k^		
Normal (≥2500 g)	15	68
Low (<2500 g)	7	32

Abbreviations: FQ, fluoroquinolone; MDR/RR, multidrug-resistant/rifampicin-resistant; SLI, second-line injectable; XDR, extensively drug-resistant.

^a^A total of 48 pregnancies were reported for 43 women; 5 women had multiple pregnancies during the study; 1 woman had twins.

^b^Countries coded to remove indirect identifier of concerned women. These include Armenia, Georgia, Haiti, Indonesia, Kazakhstan, Lesotho, Pakistan, and Peru.

^c^Patient's age at time of treatment initiation.

^d^Patient's body mass index at time of treatment initiation. Weight not available for 1 patient.

^e^Patient's human immunodeficiency virus status at time of treatment initiation.

^f^Patient's drug resistance profile at time of treatment initiation. MDR/RR, resistant to isoniazid and rifampicin; MDR/RR + SLI, resistant to isoniazid and rifampicin *and* any second-line injectable only; MDR/RR + FQ, resistant to isoniazid and rifampicin *and* any fluoroquinolone only; XDR, resistant to isoniazid and rifampicin *and* any fluoroquinolone *and* at least 1 second-line injectable.

^g^Extensive disease, 3+ smear-positives with cavitation on chest X ray. Unknown if smear results or chest X ray not available. For the 3 patients who had extensive disease, 2 were cured and 1 died.

^h^Treatment outcomes: successful, cured, or treatment completed; poor, treatment failure, lost to follow-up, or died. There were no outcomes not evaluated.

^i^Duration of MDR/RR tuberculosis treatment during pregnancy. Excludes 5 women who became pregnant after the end of treatment and 3 women whose pregnancy outcome dates were unknown.

^j^Birth outcomes for 32 fetuses from 31 continued pregnancies. Includes 1 set of twins, both live births.

^k^Birth weight unknown for 4 infants.

Most pregnancies (73%, 35 of 48) began after MDR/RR-TB treatment initiation at a median of 258 days (range, 26–668) from enrollment. Eight (17%) pregnancies began prior to starting MDR/RR-TB treatment, and 5 (10%) pregnancies occurred after completion of treatment. Of the 8 pregnancies that began prior to treatment, 4 (50%) were in the first trimester, 3 (38%) in the second trimester, and 1 (12%) in the third trimester at treatment start. For the women who became pregnant before the end of their treatment, median time of exposure to MDR/RR-TB medication during pregnancy was 82 days (range, 9–304). Bedaquiline was used in all 5 pregnancies that occurred after treatment completion, within 5 months of the last dose at a median of 123 days (range, 5–138). Taken together, 17 of 48 (35%) pregnancies were electively terminated. Of 31 pregnancies that were continued, there were 32 birth outcomes, including 1 set of twins. There were 26 (81%) live births, 4 (13%) unknown outcomes, and 2 (6%) that ended in spontaneous abortion early in pregnancy (8–14 weeks of gestation). Among 22 live births with known birthweight, 15 infants (68%) had normal birthweight (≥2500 g) and 7 (32%) had low birthweight. The twins, both live births, and 7 other babies were born preterm (30–37 weeks of gestation). During the post-birth period, 1 baby was started on DR-TB treatment and 1 baby with low birthweight died within 4 months from complications unrelated to TB. There were no maternal deaths or reported birth malformations.

## DISCUSSION

In this prospective study of pregnant patients who received second-line MDR/RR-TB regimens that contained bedaquiline or delamanid, 98% of all mothers had successful treatment outcomes, and at least 81% of continued pregnancies resulted in live births with 68% normal birthweight neonates. These TB treatment and pregnancy outcomes compare favorably with results from recent studies [13–15], including end-of-treatment outcomes reported for the overall endTB cohort [16, 17].

First-line TB drugs are safe during pregnancy, and treatment improves maternal and neonatal outcomes [18]. Meanwhile, little is known about the use of second-line TB drugs in pregnancy, and pregnant women are excluded from clinical research on which WHO recommendations are based [2, 3]. Women with TB and their neonates have a greater risk of mortality [19]. A study from Peru found high treatment success rates in both pregnant and nonpregnant women with pulmonary TB, irrespective of drug-susceptibility profiles, demonstrating that pregnant women can experience successful outcomes with appropriate treatment [20]. A South African study that compared outcomes in pregnant women who did and did not receive bedaquiline found that fetal exposure to bedaquiline in utero was associated with low birthweight (<2500 g), with no other significant differences in infant, pregnancy, or maternal treatment outcomes [14]. Another small case study from South Africa found successful maternal treatment outcomes and excellent birth outcomes in children born to pregnant women who were receiving bedaquiline, delamanid, and linezolid [21]. In light of this evidence, in their most recent update to treatment guidelines, WHO now recommends a 9-month, all-oral regimen that includes linezolid, instead of ethionamide, for pregnant women with MDR/RR-TB without documented FQ resistance [5].

In the endTB and STEM-TB studies, we counseled women and their partners on the use of contraceptives and provided resources. However, contraceptive use was not mandated, and uptake varied by personal preference, access to free contraception, and cultural barriers. In our study, 17 patients terminated their pregnancies.

While our study was small, it comprised a diverse population from 8 participating countries. The limited number of participants precluded study of the role of HIV coinfection and other comorbidities. Our study was not designed to look at in utero risk of individual drug exposure. However, we systematically collected data for all patients with close follow-up monitoring and reporting of safety events. Data on breastfeeding as well as any longer-term follow-up of infants beyond 1 year of age was not formally collected.

## CONCLUSIONS

Pregnant women who received effective MDR/RR-TB treatment regimens had excellent outcomes, and no major negative impacts were detected in their infants. These data could be accentuated by comparisons with other cohorts not affected by TB or exposed to different MDR/RR-TB treatments and identification of factors that contribute to low birthweight in babies born to mothers with MDR/RR-TB. Inclusion of pregnant women in clinical trials would facilitate generation of these data. Finally, a global registry is urgently needed to contribute to the evidence base to assist parents, clinicians, and TB programs with informed decision-making.

## Supplementary Data

Supplementary materials are available at *Clinical Infectious Diseases* online. Consisting of data provided by the authors to benefit the reader, the posted materials are not copyedited and are the sole responsibility of the authors, so questions or comments should be addressed to the corresponding author.

## Supplementary Material

ciad445_Supplementary_Data

## Notes

***Author Contributions.*** I. L. F., N. L., S. A., P. Y. K., M. F. F., and U. K. conceived and designed the study. I. L. F., N. L., S. A., M. M. A., Y. A., S. C., D. H., A. J., P. Y. K., G. L. J., N. L., N. M., G. M., E. O., S. P., F. A. P., M. R., Y. S., E. S. G., A. S., D. V. V., M. F. F., and U. K. acquired the data. I. L. F., N. L., S. A., P. Y. K., M. F. F., and U. K. analyzed and interpreted the data and wrote the manuscript. I. L. F., N. L., S. A., C. H., H. H., P. Y. K., C. D. M., M. L. R., M. F. F., and U. K. critically revised the manuscript. All authors agreed with the study's results and conclusions.

***Acknowledgments.*** The authors thank the funders, Unitaid and the National Institutes of Health, for financing the endTB and STEM-TB projects, respectively. They are indebted to the patients who participated in the studies and to the clinicians and program staff of the participating national tuberculosis (TB) programs. The authors thank staff at Partners In Health, Médecins Sans Frontières, Epicentre, and Interactive Research and Development.

***Disclaimer.*** All authors report that the endTB consortium coordinated donations of delamanid from Otsuka Pharmaceuticals to be used for treatment of some patients included in the endTB observational study and donations of bedaquiline from Janssens to be used for treatment of some patients included in the endTB observational study.
