# Supplementary material for: Pregnancy and Birth Outcomes in Patients With Multidrug-Resistant Tuberculosis Treated With Regimens That Include New and Repurposed Drugs
Source: Clin Infect Dis. 2023 Aug 22;78(1):144–8. doi: 10.1093/cid/ciad445 (PMC10810705; doi:10.1093/cid/ciad445)
Supplement: ciad445_Supplementary_Data [file ciad445_supplementary_data.docx]

Table S1: Individual treatment regimens of pregnant patients receiving MDR/RR-TB treatment including new and repurposed drugs

| Patient | Group A | Group B | Group C | Others |
| --- | --- | --- | --- | --- |
| 1 | Bdq, Lzd | Cfz | Dlm, Ipm-Cln |  |
| 2 | Bdq, Lzd | Cfz | Dlm, Ipm-Cln |  |
| 3 | Mfx |  | Ipm-Cln, PAS | Cm |
| 4 | Mfx, Bdq, Lzd | Cs | PAS | Cm |
| 5 | Lfx, Bdq, Lzd | Cs | E, Z, Am, Eto-Pto |  |
| 6 | Mfx, Bdq, Lzd | Cs | E, Z, Am, Eto-Pto, PAS |  |
| 7 | Mfx, Bdq | Cs | E, Z, Am, Eto-Pto |  |
| 8 | Lfx, Bdq, Lzd | Cs | E, Z, Eto-Pto | Cm |
| 9 | Mfx, Bdq, Lzd | Cs | E, Z, Am, Eto-Pto |  |
| 10 | Bdq, Lzd |  |  |  |
| 11 | Lfx, Bdq, Lzd | Cs | Eto-Pto |  |
| 12 | Mfx, Bdq, Lzd | Cfz |  |  |
| 13 | Mfx, Bdq, Lzd | Cfz | Dlm, PAS |  |
| 14 | Mfx, Bdq, Lzd | Cfz, Cs |  |  |
| 15 | Bdq, Lzd | Cfz | Z, Am |  |
| 16 | Bdq, Lzd | Cfz, Cs | Z, Am, Eto-Pto |  |
| 17 | Bdq, Lzd | Cfz, Cs | Z, Eto-Pto |  |
| 18 | Bdq, Lzd | Cfz, Cs | Z, PAS |  |
| 19 | Lfx | Cs | Z, Eto-Pto |  |
| 20 | Lfx, Bdq, Lzd | Cfz, Cs | Z, Eto-Pto |  |
| 21 | Bdq, Lzd | Cfz, Cs | Z, Ipm-Cln |  |
| 22 | Mfx, Lzd | Cfz, Cs | Dlm, Z, Eto-Pto, PAS |  |
| 23 | Mfx, Bdq, Lzd | Cs | Z, Am, Eto-Pto |  |
| 24 | Mfx, Bdq, Lzd | Cfz, Cs | Z, Am |  |
| 25 | Bdq, Lzd | Cfz, Cs | Z, Am |  |
| 26 | Bdq, Lzd | Cfz | Z |  |
| 27 | Bdq, Lzd | Cs | Z | Cm |
| 28 | Mfx, Lzd | Cfz | Dlm, Z, Eto-Pto | Cm |
| 29 | Bdq, Lzd | Cfz, Cs | Z | Cm |
| 30 | Lfx, Bdq, Lzd | Cs | Z, PAS | Cm |
| 31 | Bdq, Lzd | Cfz | Dlm, Z, Eto-Pto |  |
| 32 | Bdq, Lzd | Cs | Z, Ipm-Cln, Eto-Pto |  |
| 33 | Lfx, Lzd | Cs | Dlm, Z |  |
| 34 | Lfx, Lzd | Cfz | Dlm, Z |  |
| 35 | Mfx, Lzd | Cfz, Cs | Dlm, Z |  |
| 36 | Bdq, Lzd | Cfz, Cs | Dlm, Z |  |
| 37 | Bdq, Lzd | Cfz, Cs | Z, Eto-Pto |  |
| 38 | Lfx, Bdq, Lzd | Cfz, Cs |  |  |
| 39 | Lfx, Bdq, Lzd |  | Dlm, Z |  |
| 40 | Lfx, Bdq, Lzd |  | Dlm, Z |  |
| 41 | Lfx, Bdq, Lzd | Cfz, Cs |  |  |
| 42 | Lfx, Bdq, Lzd | Cfz, Cs |  |  |
| 43 | Lfx, Bdq, Lzd | Cfz, Cs |  |  |

Lfx: Levofloxacin, Mfx: Moxifloxacin, Bdq: Bedaquiline, Lzd: Linezolid, Cfz: Clofazimine, Cs: Cycloserine, E: Ethambutol, Dlm: Delamanid, Z: Pyrazinamide, Ipm-Cln: Imipenem-cilastatin, Am: Amikacin, Eto-Pto: Ethionamide-Prothionamide, PAS: *P*-aminosalicylic acid, Cm: Capreomycin
